# MicroRNAs: Emerging Novel Targets of Cancer Therapies

**DOI:** 10.1155/2015/506323

**Published:** 2015-03-19

**Authors:** Chengfeng Yang, Yiguo Jiang, Ajay P. Singh, Fumitaka Takeshita

**Affiliations:** ^1^Department of Physiology, Center for Integrative Toxicology, 4171 Biomedical Physical Sciences, Michigan State University, East Lansing, MI 48824, USA; ^2^Institute for Chemical Carcinogenesis, State Key Laboratory of Respiratory Disease, Guangzhou Medical University 195, Dongfeng Road West, Guangzhou, Guangdong 510182, China; ^3^Department of Oncologic Sciences, Mitchell Cancer Institute, University of South Alabama, 1660 Springhill Avenue, Mobile, AL 36604-1405, USA; ^4^Division of Molecular and Cellular Medicine, National Cancer Center Research Institute, 1-1 Tsukiji 5-chome, Chuo-ku, Tokyo 104-0045, Japan

MicroRNAs (miRNAs) are a large family of small noncoding RNAs (~22 nucleotide long) that negatively regulate protein-coding gene expression posttranscriptionally by interacting with messenger RNAs (mRNAs), causing either their degradation or translation inhibition. While miRNAs were first discovered as important regulators of developmental timing, subsequent studies have shown that their deregulations are critically involved in various diseases, including cancer. This is evident from the crucial roles they play in regulating a wide range of cellular processes such as cell survival, apoptosis, cell cycle progression and proliferation, cell-cell interaction, differentiation, and motility.

It has been found that miRNA expression levels are altered in all types of cancer and they play important roles in almost all aspects of cancer pathogenesis such as initiation, promotion, metastasis, and responses to drug treatment. These recognitions and other accumulating evidences clearly suggest that miRNAs can serve as novel targets for cancer therapies. Indeed, targeting abnormally-expressed miRNAs has been shown to have great potential in suppressing primary tumor growth, reducing tumor metastasis, and overcoming anticancer drug resistance. It is anticipated that miRNAs can be valuable cancer-specific targets and novel miRNA-based targeted therapies can be formulated to provide effective treatments for cancer. The purpose of this special issue is to provide readers with an overview of research findings on the roles of miRNAs in cancer development, progression, diagnosis and treatment, and how miRNA expression may be regulated. This issue will also update the readers about the challenges and possibilities of miRNAs to emerge as future cancer therapeutics.

A. L. Oom et al. first briefly review the potential value of miRNAs as cancer diagnostic markers, which is followed by the detailed discussion on the potential role of miRNAs in cancer therapy. The authors review literatures reporting the role of miRNAs in sensitizing of or mediating resistance to traditional and targeted cancer therapies. A nice summary about studies on miRNAs in combination with traditional cancer therapies is provided in a table. The authors also discuss the current studies on miRNA delivery systems. This review ends with discussion on challenges and perspectives of developing miRNAs as effective cancer therapies.

The review paper, authored by K. Otsuka and T. Ochiya, discusses the role of miRNAs in tumor development by uniquely focusing on the interplay between the classic tumor suppressor p53 and the emerging tumor regulatory miRNAs. The authors review recent findings showing both the regulation of expression of various miRNAs by p53 and the direct targeting of p53 by miRNAs. Particularly, this review summarizes up to date research findings about the critical roles of various miRNAs in regulation of cell cycle and apoptosis in the p53 pathways.

Y. Fujita, K. Kuwano, T. Ochiya, and F. Takeshita provide an in-depth review of current findings showing the potential of circulating miRNAs as diagnostic biomarkers for lung cancer. This review discusses the general mechanism of miRNA release into extracellular spaces and the impact of extracellular vesicle-encapsulated circulating miRNAs on cancer progression. Moreover, this review also summarizes current studies in detail showing the potential of miRNAs as circulating biomarkers in cancer diagnosis, particularly for lung cancer.

N. H. Phuah and N. H. Nagoor review the regulation of miRNAs by natural compounds. Recent studies have shown that natural agents such as curcumin, resveratrol, genistein, epigallocatechin-3-gallate, indole-3-carbinol, and 3,3′-diindolylmethane exert their antiproliferative and/or proapoptotic effects through the regulation of one or more miRNAs. This review summarizes current research findings about the regulation of miRNAs by above natural compounds. The review also discusses the potential of integrating natural agents with conventional chemotherapeutic drugs, thus, enhancing their efficacy.

The review article by S. K. Srivastava, S. Arora, C. Averett, S. Singh, and A. P. Singh discusses the effect of naturally occurring phytochemicals on miRNA expression. Particularly, the authors focus on the underlying molecular mechanisms of miRNA regulation by phytochemicals and their functional significance in cancer development and progression. Moreover, the translational potential of the relevant research findings is also discussed.

In closing, the guest editors of this special issue would like to thank all the reviewers for their timely and excellent review and journal management team for efficient processing of papers from submission through publication.

*Chengfeng  Yang* *Yiguo  Jiang* *Ajay  P. Singh* *Fumitaka  Takeshita*

